# Comprehensive Risk Assessment of Metals and Minerals in Seafood Using Bioaccessibility Correction

**DOI:** 10.3390/jox15030092

**Published:** 2025-06-12

**Authors:** Ștefania-Adelina Milea, Ira-Adeline Simionov, Nina-Nicoleta Lazăr, Cătălina Iticescu, Mihaela Timofti, Puiu-Lucian Georgescu, Caterina Faggio

**Affiliations:** 1 REXDAN Research Infrastructure, “Dunărea de Jos” University of Galati, 98 George Coșbuc Street, 800385 Galati, Romania; adelina.milea@ugal.ro (Ș.-A.M.); nina.condurache@ugal.ro (N.-N.L.); catalina.iticescu@ugal.ro (C.I.); mihaela.timofti@ugal.ro (M.T.); 2 Faculty of Food Science and Engineering, “Dunărea de Jos” University of Galati, Domnească Street, 800008 Galati, Romania; 3 Faculty of Sciences and Environment, “Dunărea de Jos” University of Galati, Domnească Street, 800008 Galati, Romania; 4 Department of Chemical, Biological, Pharmaceutical and Environmental Sciences, University of Messina, 98166 Messina, Italy; cfaggio@unime.it; 5 Department of Eco-Sustainable Marine Biotechnology, Stazione Zoologica Anton Dohrn, 80121 Naples, Italy

**Keywords:** seafood contamination, dietary exposure, in vitro digestion, health risk, food safety

## Abstract

Evaluating the bioaccessibility and health risks of seafood is extremely important because, although it is a significant source of vital minerals, it may also contain potentially toxic elements. This study aimed to determine the content of metals and minerals in different seafood species before and after thermal processing. Also, given the risk of overestimating the actual final concentration available in the body, a study was carried out to determine the bioaccessibility of these elements by simulating the digestion process in the gastrointestinal tract. Assessment of the potential toxic effects on consumer health in terms of exposure to heavy metals was carried out through risk analysis by Estimated Daily Intake, Hazard Index, and Cancer Risk parameters. Three bivalve mollusks, one gastropod mollusk, four cephalopod mollusks, and one crustacean species were analyzed in terms of minerals (P, S, K, Ca, and Se) and heavy metals (Cd, Pb, Ni, Cr, Fe, Zn, Co, Mn, and As) content. The lead (Pb) concentration recorded the strongest bioaccessibility increase, even reaching 100% in *P. vannamei*. Generally, the bioaccessibility of all metalloids dropped below 100%, which suggests that only a part of the amount of metal in the initially ingested sample can be absorbed by the human organism. Potassium and sulfur registered the greatest value, up to 23% for minerals’ bioaccessibility in the same samples. The highest intake rate of metals occurred after the consumption of *M. gigas*, which registered the highest Estimated Daily Intake for Cr (chromium) (0.321 mg kg^−1^ d^−1^), Cu (copper) (10.15 mg kg^−1^ d^−1^), and Zn (zinc) (12.67 mg kg^−1^ d^−1^). The Hazard Index values indicated no significant risk of poisoning. All calculated Cancer Risk scores remained below the acceptable threshold. Moreover, the Pearson coefficient revealed a positive correlation between the Hazard Index and the most abundant elements in the samples, Cr, Zn, and Cu. This study could provide a framework for evaluating both the nutritional benefits and toxicological concerns of seafood intake in public health applications.

## 1. Introduction

Since the 1970s, global fish and seafood production has seen a quadruple increase [1]. During this time, the global population has more than doubled, and the average individual consumes about twice as much fish and seafood as they did fifty years ago [2]. Seafood represents a valuable component in the human diet because it is an excellent source of minerals, vitamins, unsaturated fats, phospholipids, necessary elements, and high-biological-value proteins. Minerals are essential to human metabolism [3]. In addition to supporting the development of an organism’s bone structure, minerals from seafood can also catalyze the synthesis of other vital biological components including enzymes, hormones, and vitamins, as well as help with the maintenance of colloidal systems and pH equilibrium [4]. In particular, iron (Fe) helps the body to carry oxygen and supplies energy; calcium (Ca) is necessary for healthy bones; and magnesium (Mg) and phosphorus (P) may also control protein activity and prevent certain illnesses [5]. Apart from providing essential nutrients, fish and seafood products can also accumulate significant amounts of harmful elements, such as heavy metals [6]. A wide range of various heavy metal concentrations have been previously identified in seafood species [6]. According to Soltani et al. [7], the most common among them are arsenic (As), cadmium (Cd), nickel (Ni), lead (Pb), and mercury (Hg), which are of particular concern because of their persistence and bioaccumulation potential at trace concentrations. Due to their physicochemical properties, heavy metals are naturally occurring non-degradable contaminants that mostly originate from the earth’s crust, sediments, and waste products generated from human activity, among other sources, in the aquatic environment [8,9]. For these reasons, monitoring the levels of heavy metals is vital to the ecosystem [10]. The aquatic environment has become contaminated with many pollutants, due to industrialization and increased human activity, which may represent a health risk to humans by transfer into the food chain [11]. Urbanization has a considerable influence on water quality by importing pollutants such as fertilizers, heavy metals, sediments, and organic contaminants via runoff, altering aquatic ecosystems and threatening human health [12]. De Souza et al. [13] highlight the significance of urban pollution in aquatic ecosystems and its direct implications for food safety. Unlike other contaminants, heavy metals may be mobilized in aquatic environments and accumulate in aquatic organisms, making them hazardous, non-biodegradable, and very toxic [14]. Their toxicity can damage the kidneys, liver, brain, lungs, blood, and other essential organs while also weakening energy. Prolonged exposure ultimately leads to degenerative processes in the body, brain, and tissues that mimic illnesses, including multiple sclerosis, Parkinson’s, Alzheimer’s, and muscular dystrophy [15]. However, metals ingested through food are not equally bioavailable to the organism. A limited quantity of heavy metals found in seafood tissue may be absorbed physiologically during human consumption. This specific quantity, known as bioaccessibility, normally refers to the percentage of substances that are ingested and pass through the mouth, stomach, and gastrointestinal tract [10]. The release of a substance from consumed food is a necessary precursor for absorption and assimilation. The discharged contaminant may be partly or completely absorbed by the intestines, eventually entering the systemic circulation [16]. PTEs in seafood can bioaccumulate and change into several dangerous forms. Human risk is determined not just by exposure, but also by the amount produced during digestion and absorbed by the body. Chemical species can also influence metal bioaccessibility because of their specific properties under special digestion conditions. For example, methylmercury and inorganic arsenic, the toxic species for these both metals, are highly absorbed after the digestion process [17].

For example, HgT bioaccessibility in seaweed and lobster hepatopancreas was 3% and 4% [18], and in tuna ranges from 13% to 19% [19], whereas in sardines, tuna, and swordfish, it varies from 9% to 17% [20]. Arsenic bioaccessibility is significantly greater in whitefish, cold-water fish, and mollusks, ranging from 83% to 99% [21]. Bioaccessibility can be explored using in vitro gastrointestinal digestion. It is a cost-effective and predictable approach and it can be used as an alternative to oral bioavailability in risk assessments [22]. This method offers a substitute for animal research, which requires ethical protocols and specific infrastructure [23]. Nevertheless, a recognized validation technique for bioavailability research is still lacking. Methodological and conceptual limitations still need to be discussed despite the topic’s rising popularity. It is important to assess whether the impact demonstrated in vitro will be replicated in vivo, since other compounds such as intestinal bacteria may have an impact on the interactions observed during digestion.

The in vitro approach cannot reproduce the entire absorption and digestion process and needs to be combined and more intensively explored. Besides the aforementioned methodological considerations, variables such as diet, genetics, and gut microbiota are becoming more widely recognized as potential contributors to variation in metal bioavailability [24].

The phenomenon of heavy metals bioaccumulating in the body can be explained by the fact that they are absorbed and retained more quickly than they can be eliminated [25]. This can be demonstrated by determining a higher concentration of toxic compounds in the body than in the environment. Bioaccumulation is a hazardous process that raises concerns regarding the impact on human health and increases the transfer probability of contaminants in the food chain [25]. This idea also introduces the concept of biomagnification, which involves increasing the contaminants’ concentration as they move up the food chain. For example, the human body is likely to have higher concentrations of heavy metals than lower trophic organisms [26].

In food safety and environmental contexts “metals”, “metalloids”, “heavy metals”, and “potentially toxic elements (PTEs)” are terms used to characterize substances of concern related to potential toxicity [27]. Metals are materials characterized by strong electrical conductivity, malleability, and brightness, which readily release their electrons to produce cations [28]. Metalloids are elements with intermediate properties between metals and non-metals [29]. Metals that negatively impact the environment and organisms and have a particular density of more than 5 g/cm^3^ are commonly referred to as heavy metals [28]. The term heavy metal does not have a broadly agreed on meaning and is frequently used ambiguously to refer to dense metals, regardless of toxicity. Because of these uncertainties, the term potentially toxic elements (PTEs) is becoming more commonly used in the scientific literature to describe metals and metalloids that may cause health hazards.

The main route through which humans are exposed to heavy metals is food consumption [30]. Therefore, it is commonly accepted that seafood is the primary dietary source of heavy metal exposure. In recent years, a lot of research has been conducted on the health risk assessment of food-borne metal exposure [31]. Generally, researchers evaluated the health risk assessment of target heavy metals based on their initial levels in food matrices. Nevertheless, in most situations, food is consumed cooked. It has been discovered that food processing procedures influence (reduce or increase) the content of the elements. The washing process, for example, has been demonstrated to release a portion of arsenic from food [32]. Schmidt et al. [33] discovered that frying might reduce about one-third of the total mercury from fish. For example, baking *Porphyra* sp. increased As bioaccessibility from 87% to 100% [34], and Se bioaccessibility in cooked cod measured 61% [35]. Moreover, the total amount of ingested substances may not accurately reflect the bioavailable portion for the consumer [16]. Previous research suggests that Cd, Hg, and other trace metals are rarely completely bioaccessible.

When it comes to food safety, the European Union enforces some of the highest standards in the world. These standards are maintained due to strong regulations and the existence of specific instruments such as the Rapid Alert System for Food and Feed (RASFF), which is an open-access online portal in which member states can report risk cases for public health occurring in the food chain. The European Food Safety Authority (EFSA) provides scientific assessments and recommendations related to contaminants, including heavy metals in food, including seafood. The European Commission relies on the EFSA for the development of regulations and policies based on scientific evidence and data. At the same time, the EFSA supports ongoing monitoring and surveillance activities carried out by member states to track levels of contaminants in food. This information is crucial for risk assessments and regulatory decision-making. Thus, in April 2023, the Commission Regulation (EC) 1881/2006 was replaced by the [36], which sets new limits for contaminants in foodstuffs, including heavy metals in fish and fishery products.

In the last 20 years, the top 10 countries to issue notifications on heavy metals in different foods include Spain, Italy, and Belgium. Romania is not one of those ten countries, thus emphasizing the need to step up monitoring strategies of heavy metals in foodstuffs. At the same time, the (EU) 2023/915 has certain drawbacks when it comes to setting the maximum levels of heavy metals in fish and fishery products, because it considers the concentrations applied to the wet weight of the products. For the first time, our study proposes conducting a health risk assessment analysis related to heavy metals in human exposure, considering the bioaccessible concentration fractions following temperature treatment and digestion process.

In order to support the need for the European Union to modify the existing measures or adopt additional ones, the present study aims to shed light on the risk of the heavy metal intoxication of human consumers through seafood consumption and to support the EFSA by providing scientific data. A wide monitoring technique for evaluating potentially bioaccessible metal and mineral fractions in various seafood species relies on this research. The necessity for conducting this kind of study highlights exactly how important it is to adopt a permanent monitoring strategy regarding the metal and mineral composition in seafood and fish-based food. This study is founded on the hypothesis that integrating the bioaccessibility concept into the risk assessment study represents an objective approach to the toxicological risk associated with seafood consumption. Given the increasing consumer interest in such foods, a new upgraded approach from the classical methods is required. Thus, to achieve the study desideratum, the following objectives were set:

(a) The evaluation of mineral (P, S, K, Ca, Se) and heavy metal (Cd, Pb, Ni, Cr, Fe, Zn, Co, Mn, As) concentration levels with toxic potential from the edible tissues of several seafood species commercialized in the Lower Danube River Basin area and the selection of seafood species with the highest levels of heavy metals;
(b) The performance of the bioaccessibility study using the three-step in vitro digestion model;
(c) Health risk assessment of exposure to heavy metals through seafood consumption;
(d) The performance of a correlation analysis between the bio-accessible fraction of heavy metals in seafood and Cancer Risk in human consumers.

## 2. Materials and Methods

### 2.1. Seafood Sampling

Several seafood species were purchased from different vendors. The purchased specimens were taxonomically identified using the label information, as well as a specialized database (https://www.sealifebase.ca, accessed on 17 November 2024) (Table 1). Therefore, in our study, a total number of 9 seafood species were sampled as follows: 3 bivalve mollusks (*Magallana gigas, Mytilus galloprovincialis, Mytilus chilensis*), 1 gastropod mollusk (*Rapana venosa*), 4 cephalopod mollusks (*Octopus vulgaris*, *Dosidicus gigas*, *Uroteuthis duvaucelii*, *Amphioctopus membranaceus*), and 1 crustacean species (*Peneus vannamei*). Preliminary investigation of the samples included the evaluation of biometric measurements and biomass (total length and total weight), which are reported in Table 1. Further on, the specimens were thoroughly rinsed with ultrapure water (Simplicity^®^ water purification system) and roughly 20 g of each edible tissue (muscle tissue for all species, except for cephalopod species where samples of tentacles and head were collected) was collected and homogenized using a plastic utensil. The samples were then transferred into polyethylene bags and stored in the freezer until analysis. In order to prepare the samples for further analysis, they were subjected to a basic cooking method (5 min of frying at 160 °C).

### 2.2. In Vitro Gastrointestinal Digestion Model of Cooked Seafood

This study used a revised in vitro digestion model (INFOGEST 2.0), as described by Brodkorb et al. [37]. The analysis was carried out in triplicate. Heavy metal and mineral concentrations were calculated after the cooked seafood samples were subjected to a three-step in vitro digestion model. Overall, this procedure simulates digestion in the mouth, stomach, and small intestine. Each cooked sample was first weighed, crushed, homogenized, and then transferred to the glass where in vitro digestion took place. Meanwhile, the simulated saliva fluid containing salivary α-amylase (SSF, pH = 7), a simulated gastric fluid including pepsin from porcine mucosa and HCl (SGF, pH = 2), and a simulated intestinal fluid with NaHCO_3_ and pancreatin from the porcine pancreas (SIF, pH = 7.8), were prepared and heated to 37 °C. Firstly, one gram of the sample was diluted in the oral phase with salivary solution 1:1 (*w*/*v*) and maintained for one minute (1 g of sample + 1 mL of SSF). The obtained oral bolus was then all mixed with the gastric fluid 1:1 (*w*/*v*) and continuously stirred for 2 h at 37 °C. In the last step, the gastric chyme was mixed 1:1 (*w*/*v*) and incubated with simulated intestinal fluid for a further 2 h. To separate the supernatants and pellets, the final mixtures were centrifuged at 4500 rpm for 15 min at 4 °C using OHAUS FC5916R Laboratory Centrifuge (Parsippany, NJ, USA).

### 2.3. Heavy Metals’ and Minerals’ Bioaccessibility from Cooked Seafood Samples

The digested seafood samples were centrifuged after digestion, and the resulting supernatant was separated from the pellet component in order to determine the element composition for both fractions. In this study, the heavy metal bioaccessibility, mineral bioaccessibility (%BA), and recovery rate (% RR) were calculated using the following equations:

(1) $$\%\;\mathrm{BA}=\frac{T_{aq}}{T_{i}}\times 100$$

where T_aq_ is the total metal or mineral concentration in the aqueous phase (ng g^−^^1^) and T_i_ is the total metal or mineral concentration in cooked samples (ng g^−^^1^).

(2) $$\%\;\mathrm{RR}=\frac{T_{aq}+T_{p}}{T_{pd}}\times 100$$

where T_aq_ is the total metal or mineral concentration in the aqueous phase (ng g^−^^1^), T_p_ is the total metal or mineral concentration in the pellet (ng g^−^^1^), and Tpd is the total metal or mineral concentration in the test food before the in vitro digestion process (ng g^−^^1^).

### 2.4. Heavy Metal Analysis

Prior to the analysis, all fresh samples were digested according to the previously described protocols by [6,38]. Briefly, 0.5 g of the sample was introduced in the PTFE-TFM vessels and Suprapur^®^ nitric acid (HNO_3_ 65%) and perhydrol^®^ (H_2_O_2_ 30% EMESURE^®^) were added. A three-step digestion program was applied to mineralize the samples, using the microwave assisted Ethos™ Easy (Milestone SRL, Bergamo, Italy) equipment. After the digestion process, the resulting aqueous solution was transferred into centrifuge tubes and diluted with demineralized water (50 mL volume).

A total number of nine heavy metals were analyzed as follows: cadmium (Cd), cobalt (Co), chromium (Cr), copper (Cu), iron (Fe), manganese (Mn), nickel (Ni), lead (Pb), and zinc (Zn). The analytical technique used to quantify the target compounds in samples was the inductively coupled plasma mass spectrometry (ICP-MS) method, and the NexION 2000 (PerkinElmer Inc., Shelton, CT, USA) apparatus was employed for the analysis. The technical performance of the instrument was verified before each working session by conducting the standard performance check.

An ICP-MS multi-element standard solution IV 23 element in diluted nitric acid (1000 mg L^−^^1^) was used to construct the calibration curve in 5 points (10, 20, 30, 40, and 50 µg L^−^^1^). Before the working session, the performance of the ICP-MS equipment was checked using the NexION Setup Solution and SmartTune™ program to ensure that all technical parameters (gas flow, torch alignment, etc.) were within the accepted range. The limit of detection (LOD) for the analyzed elements is as follows: Cd (LOD = 0.00006 µg L^−^^1^), Co (LOD = 0.000006 µg L^−^^1^), Cr (LOD = 0.00005 µg L^−^^1^), Cu (LOD = 0.00003 µg L^−^^1^), Fe (LOD = 0.0001 µg L^−^^1^), Mn (LOD = 0.00005 µg L^−^^1^), Ni (LOD = 0.00006 µg L^−^^1^), Pb (LOD = 0.00001 µg L^−^^1^), and Zn (LOD = 0.0001 µg L^−^^1^). The results were verified using certified reference material from the Joint Research Centre for mussel tissue (ERM^®^-CE278k, sample no. 0914). The reference material was subjected to the same processing steps and analyzed like the other samples. The recovery rates were calculated and are presented in Table 2.

### 2.5. Total Reflection X-Ray Spectrometry Analysis

Approximately 1.7 mL from the mineralized samples was mixed with 0.2 mL polyvinyl alcohol (PVA) solution (3 g L^−^^1^) and vigorously agitated. Subsequently, Ga was added into the mixtures as the internal standard in concentrations between 100 and 5000 µg L^−^^1^ and again homogenized well. Afterwards, 10 µL of the obtained mixtures was transferred onto the center of the quartz carriers and dried at 50 °C for 5 min using a hot plate.

TXRF analyses were performed employing a S4 T-Star spectrometer (Bruker Nano GmbH, Berlin, Germany), equipped with a Molybdenum X-ray tube (50 W) operating at 50 kV and 1000 µA. Each measurement was carried out in triplicate under ambient air conditions, with a duration of 1000 **s** per replicate. Using the TXRF method, elements such as P, S, K, Ca, Se, and As were identified and quantified.

The quantification of the elements from the samples was based on the sensitivity and concentration of the internal standard using the following equation:

Ci=CIS·Ni·SIS/NIS·Si

where Ci = element concentration, CIS = internal standard concentration, Ni = element net count rate, NIS = internal standard net count rate, Si = element sensitivity factor, and SIS = internal standard sensitivity factor.

The recovery rates of K, Ca, Se, and As were previously presented by Lazăr et al. [39].

### 2.6. Health Risk Analysis

In order to identify the possible human intoxication risks associated with seafood consumption of heavy metals, health risk assessment analysis was performed. Within the analysis, the concentrations of heavy metals detected in the liquid fraction after performing in vitro digestion were used due to the fact that this is the quantity of metals reaching the tissues.

#### 2.6.1. Non-Carcinogenic Parameters

For the health risk assessment analysis, the first calculated parameter is the Estimated Daily Intake (EDI), which estimates the ingested quantity of a substance from a certain foodstuff on a daily basis. In our case, the EDI of heavy metals from seafood was calculated according to Bassey, by applying the following equation [40].

$$EDI(mg/kg/day)=\frac{EF\times ED\times IR\times C}{BW\times ATn}$$

where

EF = Exposure frequency (365 d/year);
ED = Exposure duration—adult average lifespan in Romania (74.2 years) according to the European Commission [41];
IR = Ingestion rate (22.22 g per person/d) according to the European Market Observatory for Fisheries and Aquaculture Products, 2022;
C = Concentration of heavy metals in seafood (mg kg^−^^1^);
BW = Average body weight of Romanian adults (78.65 kg) according to World Data;
ATn = Average exposure time (365 d/year × ED).

The next step in the health risk analysis was to compute the target hazard quotient (THQ) index, which considers the concentration of heavy metals in edible tissues and the reference dose (tolerable level) known to not cause toxic effects. If the scores for the THQ are higher than 1, it is considered that a moderate or high risk of intoxication has manifested. The THQ was calculated according to [42], as follows:

$$THQ=\frac{EF\times ED\times IR\times C}{RfD\times BW\times ATn}\times 10^{-3}$$

where

EF = Exposure frequency (365 d/year);
ED = Exposure duration (74.2 years);
IR = Ingestion rate (22.22 g/d);
C = Metal concentration in seafood (mg kg^−^^1^);
RfD = Oral reference dose for each metal (mg kg^−^^1^ d^−^^1^);
BW = Average body weight (78.65 kg);
ATn = Average exposure time for non-carcinogens (365 d/year × ED).

The oral reference doses (RfDs) were considered as follows: 0.001 mg kg^−^^1^ d^−^^1^ for Cd, 0.0035 mg kg^−^^1^ d^−^^1^ for Pb, 0.007 mg kg^−^^1^ d^−^^1^ for Fe, 0.014 mg kg^−^^1^ d^−^^1^ for Mn, 0.003 mg kg^−^^1^ d^−^^1^ for Cr, 0.04 mg kg^−^^1^ d^−^^1^ for Cu, 0.011 mg kg^−^^1^ d^−^^1^ for Ni, 0.03 mg kg^−^^1^ d^−^^1^ for Co, and 0.3 mg kg^−^^1^ d^−^^1^ for Zn [43,44].

Further on, the Hazard Index (HI) was calculated in order to highlight the health risks generated by exposure to the mixtures of heavy metals. Thus, the HI is computed as the sum of the THQ [44]:

$$HI=\sum_{i=1}^{n}{\left(THQ\right)}_{i}$$

The resulting scores are interpreted in the same way as the THQ [45].

#### 2.6.2. Carcinogenic Indicators

Human exposure to trace levels of carcinogenic compounds can trigger the development of cancer at some point during the lifetime. Thus, carcinogenic risk assessment analysis is undertaken by calculating the Target Cancer Risk (TR) as follows [46,47,48]:

$$TR=\frac{C\times IR\times 10^{-3}\times CPSo\times EF\times ED}{BW\times ATc}$$

where

C = Heavy metal concentration in edible tissue (mg kg^−^^1^);
IR = Ingestion rate (22.22 g per person/d);
CPSo = Cancer potency slope oral (mg kg^−^^1^ BW d^−^^1^) [49,50];
EF = Exposure frequency (365 d/year);
ED = Exposure duration (74.2 years);
BW = Average body weight (78.65 kg);
ATc = Average exposure time for carcinogens (365 d/year × ED).

The TR was calculated only for the heavy metals with carcinogenic toxicity, such as Cd, Cr, Ni, and Pb.

### 2.7. Statistical Analysis

The results for the heavy metal and mineral concentrations were presented as the average ± standard deviation to highlight the uncertainty of the data. Even though the sample size analyzed for the concentrations of heavy metals and minerals of each taxonomic group is considered adequate (n = 10), further bioaccessibility analysis was carried out only using the samples from individuals that registered the highest values, which may be considered a relatively small sample size. Nonetheless, this sample size may be considered suitable for a preliminary analysis. At the same time, it is important to mention that in aquatic environments, heavy metals will often accumulate in specific areas as “hot spots” based on hydromorphological and hydrochemical characteristics. Thus, considering this, calculating the average concentration of elements per taxonomic group might not be representative.

The relationship between heavy metals and minerals in the tissues of seafood was evaluated based on the correlation analysis and principal component analysis (PCA). The Pearson coefficient was employed to measure the correlation significance. The statistical tests were carried out using Origin Pro software, version 10.2 (OriginLab Corporation, Northampton, MA, USA) and Exploratory software, version 8.3 (Exploratory Inc., Redwood, CA, USA).

### 2.8. Chemicals and Reagents

The enzymes were obtained from Sigma-Aldrich and included salivary α-amylase, porcine pepsin, and porcine pancreatin. The following used reagents were of analytical or reagent grade: Trizma Base, hydrochloric acid, sodium carbonate, polyvinyl alcohol (Sigma-Aldrich, Schnelldorf, Germany), Gallium (Ga) standard solution (PlasmaCAL, SCP Science, Baie-d’Urfé, QC, Canada), 30% hydrogen peroxide (Merck, Darmstadt, Germany), and 65% nitric acid (Merck, Germany).

## 3. Results

### 3.1. Heavy Metals in Different Seafood Species

In the first step, nine seafood species were analyzed by quantifying the concentrations of metals in the fresh samples.

The concentration of heavy metals registered the same trend for accumulation in the tissues of the analyzed seafood species (Table 3).

It has been observed that Fe registered the highest concentration levels in all species, except for the *M. gigas* (26.13 µg g^−^^1^) and the tentacle of the *O. vulgaris* (12.23 µg g^−^^1^). At the opposite pole, the lowest concentration level of metals in all analyzed species was registered for Co, except for *P. vannamei*, which accumulated Cd the least (0.015 µg g^−^^1^ in pretreated samples and 0.013 µg g^−^^1^ in refrigerated samples).

In bivalve mollusks, the highest concentration of Cd was noted in *M. chilensis*—C1 (from Chile Aquaculture, which was previously pre-boiled), while the lowest was noted in *M*. *galloprovincialis*_BS (from fishing in the Black Sea). Significant high concentrations of Cu (23.28 µg g^−^^1^) and Zn (96.94 µg g^−^^1^) were recorded in the edible tissue of *M. gigas*. The highest levels of Fe (77.80 µg g^−^^1^) were recorded in the gastropod *R. venosa*. Regarding the heavy metal content in cephalopods, the results highlighted a value above the maximum level set by the European Union (Table 4) in the case of Pb concentration in *U. duvaucelli* tissue (3.02 µg g^−^^1^). The common octopus (*O. vulgaris*) has shown a tendency to accumulate higher levels of Cd (0.72 µg g^−^^1^), Cu (8.59 µg g^−^^1^), and Fe (20.37 µg g^−^^1^) in the head part of the body compared to the tentacles.

The levels of Co (0.035 µg g^−^^1^), Cr (0.640 µg g^−^^1^), Fe (27.31 µg g^−^^1^), Ni (0.48 µg g^−^^1^), and Zn (7.88 µg g^−^^1^) were higher in the tissues of processed *P. vannamei* than in the raw samples.

### 3.2. Heavy Metal Bioaccessibility and Recovery Rate of Cooked Seafood Samples

**Figure 1:**
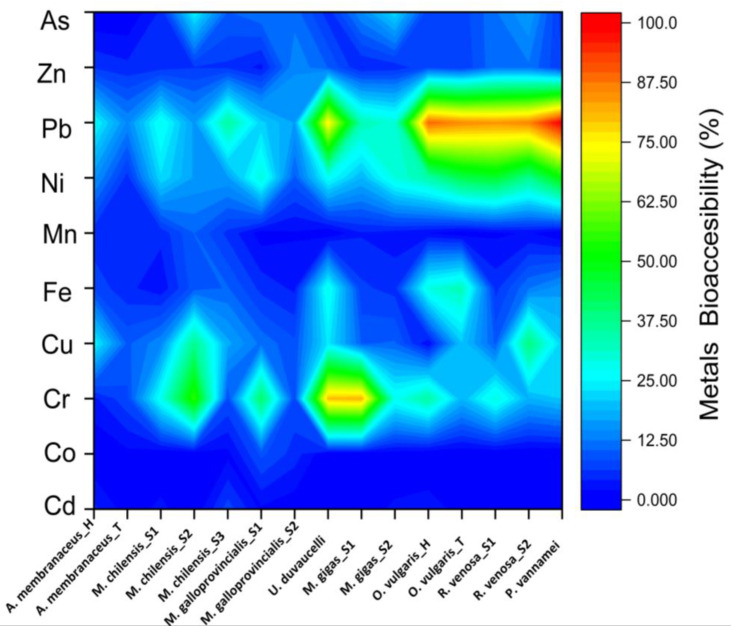
Heavy metal bioaccessibility of cooked seafood samples.

The results obtained in this study regarding the bioaccessibility values for each metal of each individual sample are presented in Figure 1.

It seems that lead (Pb) recorded the strongest increase, even reaching 100% in the *P. vannamei* sample and over 80% in the case of *O. vulgaris* (for both components—head and tentacles), and for the two analyzed specimens of *R. venosa*. Also, very high percentages were observed in the case of chromium (Cr) for a specimen belonging to *M.gigas* and *U. duvaucelli*. At the opposite pole, with percentages of bioaccessibility lower than 1%, we can mention Mn from the tentacles of *O. vulgaris_T*, Cd from *M. galloprovincialis* and the tentacles of *A. membranaceus_T*, and one specimen belonging to *M. chilensis*. Similarly, Co from *M. gigas* fell into the same trend. Moreover, the last-mentioned metal was absent in terms of bioaccessibility from most of the samples. A global profile of the analyzed seafood shows that *P. vannamei* is the most contaminated sample regarding Pb and Ni, and that the second specimen of *M. chilensis* is regarding Mn and As. Most values below 10% for each individual sample can be observed in the case of Cd, Co, and Mn.

**Figure 2:**
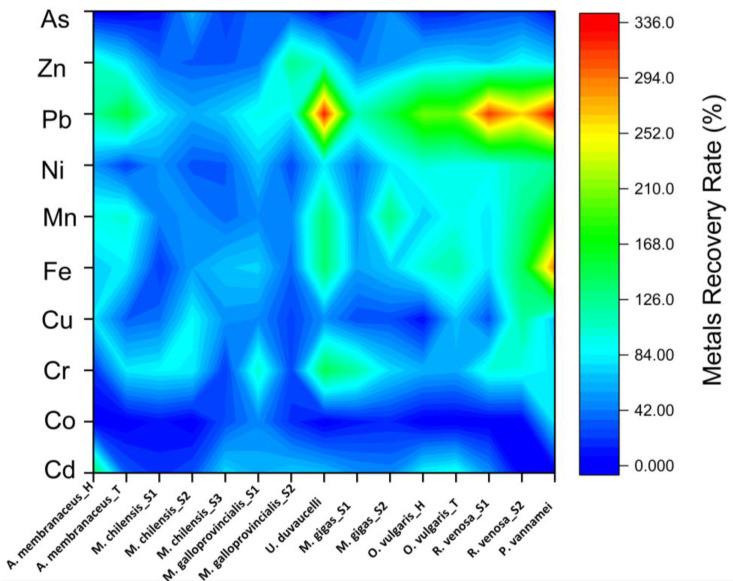
Recovery rate of metals from cooked seafood samples.

Furthermore, it is very important to know exactly what percentage of these metals can be absorbed in order to draw up clear recommendations regarding the amount of seafood that could be consumed without endangering the consumer’s health. Considering this, the recovery rate was calculated for each element and sample (Figure 2).

As expected, the maximum numbers for the recovery rate were recorded for lead, often exceeding 100% values. These results are in perfect correlation with the bioaccessibility rate, and could be explained by the fact that high lead concentrations were not determined in the initial thermally treated sample, but only after simulating in vitro digestion. It seems that the gastrointestinal parameters facilitate the detection of Pb. The observation that iron displays greater percentages in the case of the recovery rate than in the case of bioaccessibility leads to the hypothesis that a substantial amount of it is excreted, being present more in the solid component of the digested phase. Again, the smallest recovery rate was observed in the case of Co, where certain species such as *R. venosa*, *O. vulgaris*, and *U. duvaucelli* recorded 0 values. Except for *P. vannamei*, the rest of the samples presented low recovery rate values, up to 50%.

### 3.3. Mineral Concentrations in Seafood Samples

Table 5 displays the chemical elements found in the analyzed seafood samples using the TXRF technique.

### 3.4. Mineral Bioaccessibility and Recovery Rate of Cooked Seafood Samples

**Figure 3:**
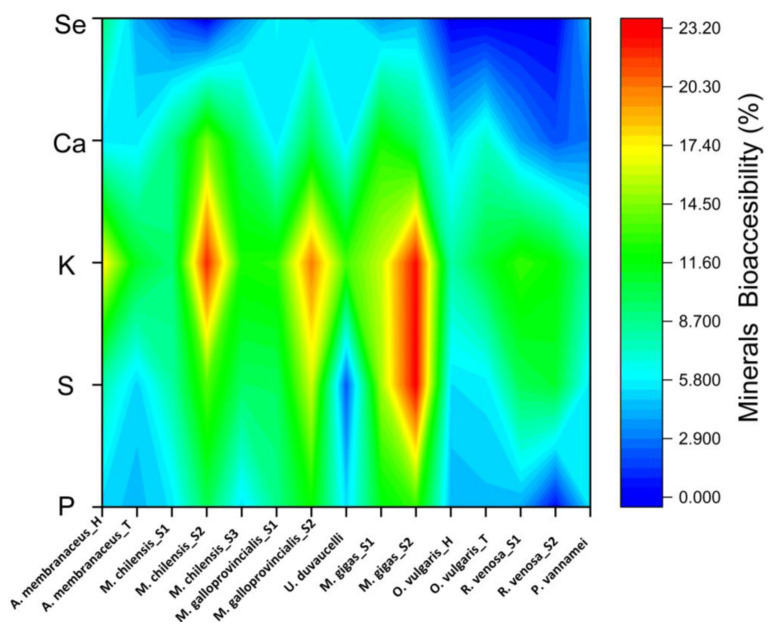
Mineral bioaccessibility of cooked seafood samples.

In this research paper, minerals’ bioaccessibility was calculated, and it is shown in Figure 3.

When the proportion of bioaccessible minerals is calculated, potassium has the greatest values, ranging from 8.00% (in the case of *P. vannamei*) to 22.79% (in the case of a specimen from *M. gigas* and 22.19 for a specimen of *M. chilensis*). The next element is sulfur, which has a bioaccessibility percentage of 23.12% in the same sample where the highest rate of potassium bioaccessibility was found. Unfortunately, selenium was missing in 5 of the 15 analyzed samples. However, a percentage of only 9% of the total existing in the sample subjected to in vitro digestion was found in the *A. membranaceus*_H sample. Generally, the samples that presented the lowest values of mineral bioaccessibility belonged to *O. vulgaris*, both head and tentacles. The bioaccessibility values for P varied between 6 and 20%. The calcium could only be calculated in one sample and recorded a value of 5% bioaccessibility and a relevant value of 50% for Zn.

**Figure 4:**
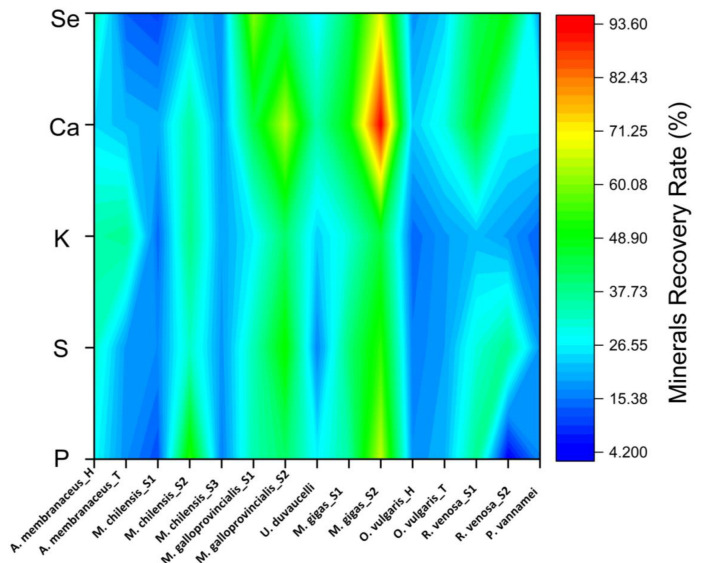
Mineral recovery rate of cooked seafood samples.

The results reported for the recovery rate of each element for each individual sample are shown in Figure 4.

The maximum recovery rate of 93.47% was calculated for Ca, and also in the case of a specimen from the species *M. gigas*. In general, all samples presented high values for the calcium recovery rate, ranging from 20.16% to 94%.

### 3.5. Health Risk Analysis

The health risk assessment analysis was performed by considering the liquid fraction obtained after digestibility, because this is the end concentration that reaches the tissues and accumulates to generate toxic effects. The highest intake rate of metals occurs after the consumption of *M. gigas*, which registered the highest EDI for Cr (0.321 mg kg^−1^ d^−1^), Cu (10.15 mg kg^−1^ d^−1^), and Zn (12.67 mg kg^−1^ d^−1^) (Table 6). The lowest risk of exposure to Cd (EDI = 0.0003 mg kg^−1^ d^−1^) and Cu (EDI = 0.0764 mg kg^−1^ d^−1^) occurs by consuming *M. galloprovincialis* (Aquaculture, Spain), while the lowest risk for exposure to Ni (EDI = 0.04 mg kg^−1^ d^−1^) and Zn (0.20 mg kg^−1^ d^−1^) occurs by consuming the cephalopod *U. duvaucelli* (Table 6). From all the analyzed species, the highest risk of Pb exposure (EDI = 0.051 mg kg^−1^ d^−1^) occurs by consuming *A. membranaceus* (the head), while the highest risk of Cd exposure (EDI = 0.04 mg kg^−1^ d^−1^) occurs from the consumption of bivalve *M. chilensis* (Table 6).

In the case of the Cancer Risk (TR) (Table 7), all scores were below 10^−^^6^ E, which is the threshold upper limit for oncogenic metals [51].

### 3.6. Correlation Between Analyzed Elements

**Figure 5:**
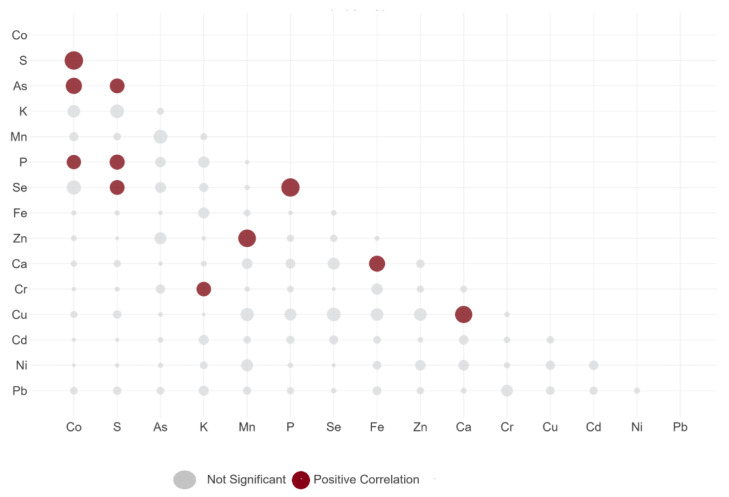
Correlation matrix between analyzed elements in the liquid fraction.

The correlation analysis (Figure 5) between elements in the liquid fraction revealed a significant positive correlation (*p* < 0.01) between S-Co, As-Co, P-Co, As-S, P-S, Se-S, K-Cr, Mn-Zn, P-Se, Fe-Ca, and Ca-Cu.

**Figure 6:**
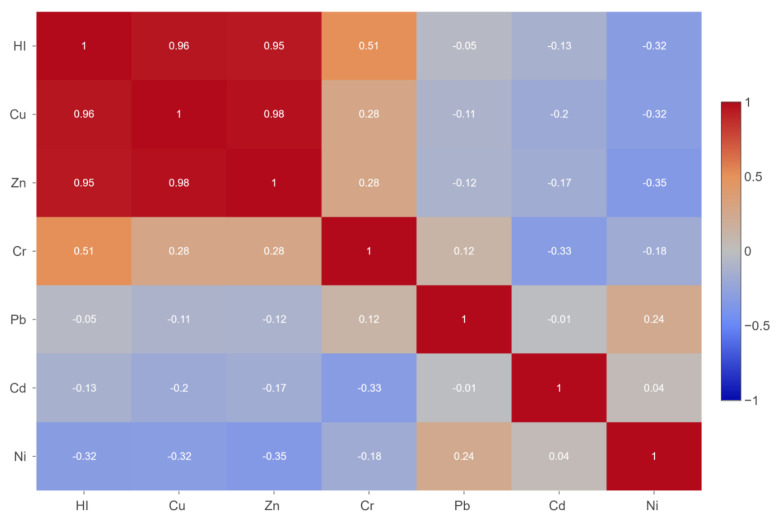
Correlation matrix between metals in the liquid fraction and Hazard Index.

The Pearson coefficient revealed a positive correlation between HI-Cr (r = 0.51), HI-Zn (r = 0.95), and HI-Cu (r = 0.96) (Figure 6). However, only the relationship between HI-Cu and HI-Zn is considered significant based on the *p* value (*p* < 0.01).

**Figure 7:**
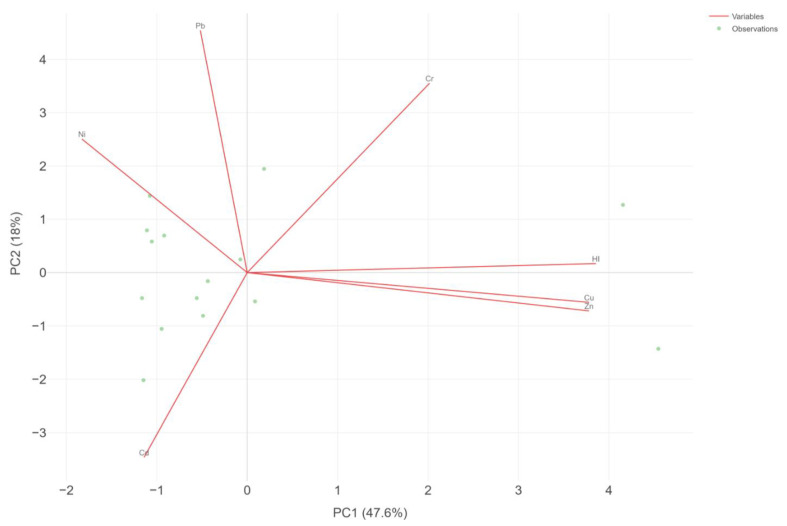
Principal component analysis between HI and heavy metals in the liquid fraction after seafood digestion.

The PCA performed for the HI and heavy metal concentration levels in the liquid fraction after digestion highlighted two major components, with an eigenvalue higher than 1. These components explain more than 63% of the data variance in the data set (Figure 7). A high correlation degree was shown between HI and Cu and HI and Zn, a relationship previously highlighted by the Pearson coefficient. This phenomenon could indicate that these metals (Cu and Zn) represent the most important fraction in determining the Hazard Index. This may be due to their high concentrations compared with the rest of the metals that are present only in trace amounts. In our study, we have obtained a range for the Cu concentration in the muscle tissue of the analyzed species between 0.58 µ/g and 23.29 µ/g, while for Zn, we obtained concentrations in the range between 5.05 µ/g and 96.94 µ/g. The abundance of Cu and Zn in invertebrates, compared to trace metals, has also been reported in a study conducted by Xia and Liu, registering concentrations of Cu in the range of 2–635 µ/g and Zn in the range of 78–491 µ/g in the tissues of freshwater mussels *Cristaria plicata* [52].

## 4. Discussion

### 4.1. Heavy Metals in Different Seafood Species

Further on, the analysis was continued by selecting the seafood specimen that registered the highest levels of metals in their fresh state, and digestibility analysis was performed considering the effect on human health.

### 4.2. Heavy Metals Bioaccessibility and Recovery Rate of Cooked Seafood Samples

Bioaccessibility refers to the amount of a contaminant that is soluble in the gastrointestinal environment and may be available for human intestinal absorption after the digestion process [8]. In this study, the bioaccessibility of heavy metals from nine types of selected cooked seafood was investigated. Metal species and speciation, as well as organic or inorganic elements that coexist in the matrix and may operate to sequester metals, are examples of variables that impact metal bioaccessibility [23]. Taking into account the European eating habits related to the fact that seafood is only consumed cooked, the bioaccessibility of heavy metals was calculated only on the thermally treated samples.

In general, the bioaccessibility of all metals analyzed in this study dropped below 100%, which suggests that only a part of the amount of metal in the initially ingested sample can be absorbed by the human organism. Previous investigations (*Regulation (EU) No 1151/2012*, [53]; Alves et al. [54]) have also shown that several metals are not completely bioaccessible. The initial metal concentration would be less realistic for estimating health risk assessment. Assessing the health risks of exposure to metals from seafood based on bioaccessibility is crucial [38]. In addition to going through extreme conditions in the gastrointestinal tract, the fact that the samples were cooked before being subjected to the in vitro digestion process had a major influence on the decrease in the bioaccessibility of the studied metals. Table 8 summarizes and centralizes the data identified in the specialized literature regarding the bioavailability of several metals after the digestion process.

Liao et al. [56] selected some seafood samples to determine the metal(loid) bioaccessibility of raw and cooked seafood. They stated that As was highly bioaccessible in raw seafood (87 to 99%), while Pb and Ni bioaccessibility was around 60 to 80%, and Cu presented an average of 71.5%. Cr had a wide variety regarding its bioaccessible fraction between all samples and recorded values from 20 to 88%. The same authors studied the bioaccessibility after various cooking methods and reported a decrease in As of 3–35% and of Cd, Pb, Ni, Cu and Zn by 30.9%, 30.7%, 25.7%, 17.6%, and 22.4%, respectively. Heat treatment can alter the nutritional compotion of seafood and affect the dissolution of metals [60]. Metals, including metalloid As, have a strong affinity for sulfhydryl groups in peptides and proteins. Heat disables proteins and breaks links with seafood proteins, making solubilization less difficult [61,62]. It seems that two processes affect the bioaccessibility of metals in seafood after cooking: protein denaturation and the release of soluble or volatile compounds into the cooking solutions or the air [56]. In study conducted by Chen et al. [23], after USEPA digestion, the bioaccessibilities of As and Cd were smaller than 9% and 16%, respectively, but the bioaccessibility of Cd ranged from 42 to 80%. On the other hand, gastric digestion generated around 10% for As, but the bioaccessibility of Cd ranged from 40 to 70%. The UBM-GI assay revealed a significant reduction in the bioaccessibility of Cd and Pb during gastrointestinal digestion (<17% and <1.5%, respectively), compared to gastric digestion exclusively. Meanwhile, Chai et al. [8] declared that heavy metal bioaccessibility in shellfish was highest for Cr (61.86%), followed by As (60.44%), Pb (55.74%), Cu (46.83%), Zn (28.16%), and Cd (24.99%). In another study, Gedik (2018) [10] analyzed metal bioaccessibility in the edible tissues of rapa whelk, and the values varied between 24.27 and 57.14% for Cr, 17.47 and 32.43% for Mn, 70.97 and 83.56% for Cu, 58.21 and 71.12% for Zn, 61.58 and 79.46% for Cd, 41.06 and 63.12% for Pb, and 53.26 and 71.48% for Ni. Generally speaking, different aquatic organisms have different amounts of metals accumulating over time. These disparities are caused by metals’ varying affinity in tissues, as well as differing rates of absorption, deposition, and excretion. Metal accumulation in seafood and fish is impacted by pollution and can vary amongst species living in the same body of water [63].

In a study conducted by Gedik et al. [10], the recovery percentages for the metals from rapa whelk recorded 96.35–106.12% percentages. In cooked seafood, Liao et al. [56] displayed recovery rates of the target metal(loid)s ranging from 86 to 112%.

### 4.3. Mineral Concentrations in Seafood Samples

This analysis highlighted the presence of P, S, K, Ca, and Se as microelements essential for the human body, but also the presence of As, a known toxic compound. As a naturally occurring metalloid element, arsenic is generally found in the air, earth’s crust, groundwater, and reaching into food products, especially in crustaceans and shellfish in varying concentrations [64].

In the analyzed seafood samples, P concentrations with values between 0.26 ± 0.01 and 2.94 ± 0.04 mg g^−1^ FW were also observed. The highest concentrations were obtained for the mussels. The Ca concentrations found in the seafood samples were in the range of 0.63 ± 0.01 and 2.16 ± 0.01 mg g^−1^ FW. Following Ca and P, the S is the most widespread mineral element in the human body. Humans absorb sulfur from their daily diets, primarily from proteins. However, only two of the twenty amino acids that are often found in proteins (methionine and cysteine) contain sulfur [65]. The S concentrations found in the seafood samples were in the range of 1.02 ± 0.05 and 6.56 ± 0.35 mg g^−1^ FW. The K, another essential microelement, presented concentrations between 0.10 ± 0.00 and 6.50 ± 0.01 mg g^−1^ FW, while Se concentrations were in the range of 0.13 ± 0.00 and 1.68 ± 0.07 µg g^−1^ FW.

Minerals are key nutrients that animals require from their food. Essential dietary nutrients are required for health and development. Non-essential elements, also known as potentially toxic elements (PTEs) cannot serve a direct nutritional function, but can be present in the diet and impact both human and animal nutrition [66]. It is already known that certain minerals can reduce the absorption or toxicity of PTEs [67]. Many investigations in animals and people have demonstrated that a lack of critical minerals such as zinc, calcium, or iron may result in increased absorption and toxicity of Cd and Pb [68]. Zinc intake has been reported to alleviate the oxidative stress caused by Cd and Pb exposure; selenium administration is protective against Cd and Pb toxicity; and iron competes with Cd for access to intestinal metal transporters [68]. Furthermore, antioxidants such as selenium can help to combat the oxidative stress induced by heavy metals [69].

### 4.4. Mineral Bioaccessibility and Recovery Rate of Cooked Seafood Samples

Because seafood meat is one of the most comprehensive foods and offers both quantity and quality of nutrients, it is becoming more and more popular among those who consume meat. Bastías et al. [70] found that an average 100 g portion of seafood contains about 50% of the necessary daily protein consumption, 10–20% of minerals, varying amounts of water-soluble vitamins, and a significant amount of liposoluble vitamins A, D, and E. Minerals are essential micronutrients that contribute to human health by participating in enzymatic processes and nutritional anabolism [71]. Mineral bioaccessibility information is fundamental when considering a dietary item as a potential mineral fortifier.

Wang et al. [72] recovered minerals from fish side streams using pressurized liquid extraction and obtained a positive effect on Ca, Zn, and Se bioaccessibility. However, a positive effect was not observed in all cases; for example, the bioaccessibility of Mg, Ca, and Fe was low in all sole extracts. De la Fuente et al. [73] analyzed the mineral bioaccessibility of the protein hydrolysates from salmon and mackerel backbones and heads.

Even though selenium was not detectable as a percentage of bioaccessibility in certain samples, it was recovered due to its high concentration in the digesta pellet in all analyzed samples. In conclusion, the predominantly higher recovery rate compared to the bioaccessibility value from the cooked seafood samples reveals that although high concentrations of minerals are ingested, they do not end up being absorbed by the human body because they are excreted.

### 4.5. Health Risk Analysis

The provisional tolerable daily intake for Cd was previously established by the Joint FAO/WHO Expert Committee on Food Additives (JECFA) at 0.00089 mg kg^−1^ d^−1^. The scores of EDI that were above the recommended values are marked in Table 6 in a red color, and it can be observed that five out of the total analyzed species present a risk of generating Cd intoxication in human consumers. Furthermore, the JECFA concluded that mollusks are among the main foods that account for 40–85% of the total mean dietary exposure to Cd, a fact which is confirmed in our present study as well [74]. Cd exercises cytotoxic effects, and it is involved in creating oxidative stress in the human body by generating reactive oxygen species, such as hydroxyl radicals (OH^-)^ and hydrogen peroxide (H_2_O_2_) [75]. Moreover, Cd is a well-known human carcinogen, contributing to the development of lung cancer in human populations [76].

Even though Cu does not generate oncogenic effects in humans, and adults can tolerate a concentration as high as 2–3 mg/day, which transposes into a provisional maximum daily intake of 0.5 mg kg^−1^ BW, exposure to this metal can contribute to Wilson’s disease [77]. As can be observed in Table 6, the EDI for *M. gigas* and *R. venosa,* both mollusk species, surpasses the recommended tolerable daily intake of Cu.

A tolerable daily intake of lead cannot be established, since exposure to this metal generates extremely hazardous effects. According to the JECFA, children who take in a Pb concentration of 0.0035 mg kg^−1^ d^−1^ exhibit an IQ decrease of at least three points [74].

The tolerable daily intake of Ni was established by the World Health Organization at 0.013 mg kg^−^^1^ d^−^^1^ [78]. The scores related to the Ni Estimated Daily Intake from Table 6 are above the WHO recommendations for all analyzed species. Nickel is a known carcinogen for humans, and exposure to it through inhalation has been associated with lung cancer [79]. At the same time, the European Food Safety Authority (EFSA) stated that developing cancer through dietary exposure to Ni is unlikely.

The trivalent form of chromium (Cr(III)) is an essential trace element involved in human metabolism, while the hexavalent form (Cr(VI)) is classified as an oncogenic metal [80]. In foods, chromium is present mainly in the trivalent form. The tolerable daily intake of Cr(III) is 0.3 mg kg^−^^1^ d^−^^1^ [80]. As can be observed in Table 6, the highest risk of Cr intoxication occurs through the consumption of bivalve mollusks such as *M. chilensis* and *M. gigas*.

Excess levels of zinc have been linked to several neurodegenerative diseases such as Parkinson’s and Alzheimer’s [81]. The JECFA established a provisional maximum tolerable daily intake of Zn between 0.3 and 1 mg kg^−^^1^ d^−^^1^ BW [74]. The results obtained from this study related to the Zn Estimated Daily Intake highlight that those consuming bivalve mollusks such as M. galloprovincialis and M. gigas have a higher risk of Zn intoxication (Table 6).

The THQ describes the coefficient between the tolerable concentration of a metal at which no negative effects are generated and the concentration at which the population is exposed [82]. The THQ values (Table 6) obtained in the present study follow the same trend as the EDI and are significantly lower than the ones obtained by Traven et al. [82] and [83]. This is due to lower seafood consumption in Romania. As can be observed in Table 6, the HI values are significantly lower than 1; thus, it can be concluded that the mixture of analyzed heavy metals from the consumption of seafood poses no intoxication risk.

### 4.6. Correlation Between Analyzed Elements

The correlation within the study can indicate the entrance pathways of heavy metals in seafood organisms through ion pumps such as Ca and K pumps. The mathematical relationship between iron and calcium can be explained by the fact that some metal transporters (DMT1) can carry divalent cations, including Fe^2+^ and sometimes Ca^2+^, under specific conditions [84]. Strong inter-correlations between metals in aquatic organisms may indicate a common source of occurrence [85].

Also, regarding the strong correlation between P-Co and S-Co, the selectivity of cobalt towards phosphates and sulfates has been pointed out previously [86]. For instance, cobalt sulfate has a higher solubility compared to elemental Co, and it has been estimated that approximately 20% of Co present in seawater is in the form of CoSO_4_ [87]. Thus, we can speculate that the cobalt accumulates in seafood bonded to P and S.

Another strong positive correlation was identified between Ca-Fe and Ca-Cu. A general opinion was drafted that Ca inhibits the absorption of Fe [88]. However, this effect is not located in the gastrointestinal tract [89]. Even further, there are reports of the interaction between Ca and Fe in the gastrointestinal tract, depending on the type of calcium salt present [90]. The same was observed in the case of the interaction between Ca and Cu [91].

The strong positive correlation between P and Se can be attributed to the synthesis metabolism of selenoprotein, a process in which selenophosphate is an important intermediate [92,93].

A strong positive correlation between Zn and Cu was noted (r = 0.98), which is an interesting observation considering that, previously, the JECFA concluded that high concentrations of Zn can generate decreased levels of Cu. The correlation analysis (Figure 6) demonstrates that this effect does not occur in the GI tract. The positive relationship between these two metals highlights their competing behavior and that they use the same pathways to enter the bloodstream.

## 5. Conclusions

This study first evaluated the concentration levels of potentially toxic heavy metals in the edible tissues of several raw seafood species. The samples that were previously processed (removal of shells and viscera and temperature treatment) tended to have higher levels of heavy metals. Second, the bioaccessibility of these heavy metals and minerals after the cooking process was assessed using a three-step in vitro gastrointestinal digestion model. The lead (Pb) showed the strongest increase, and the potassium had the greatest values in terms of bioaccessibility for many of the analyzed seafood species. Overall, the bioaccessibility values usually stayed lower than 100%, indicating that only certain amounts of ingested metals were absorbed by the human organism. Third, the values associated with the Estimated Daily Intake of metals from seafood consumption were above the provisional tolerable daily intake recommended by the JECFA. However, due to the low seafood consumption in Romania, all scores for the health risk assessment analysis were below the risk threshold. This study suggests that there is no risk of intoxication with heavy metals by consuming the analyzed seafood species. The target hazard quotient data confirmed that there is no considerable risk of heavy metal intoxication. Finally, correlation and main component assessments of bioaccessible heavy metals and Cancer Risk identified copper (Cu) and zinc (Zn) as significant contributors to the Hazard Index and possible health risk, pointing out the relevance of bioaccessibility in risk assessments.

These results provide a complete framework for assessing both the dietary benefits and toxicological concerns related to seafood intake, emphasizing the importance of bioaccessibility in food safety investigations and public health risk management.

## Figures and Tables

**Table 1:** Taxonomic identification of seafood species and specimen biometric measurements (expressed as mean ± standard deviation).

Common Name	Scientific Name	Total Length/Height * (cm)	Total Weight (g)	Origin
Giant cupped oyster—b (n = 10)	*Magallana gigas* (Thunberg, 1793)	9.9 ± 0.3	74.21 ± 10.88	Aquaculture, France
Purple whelk—b (n = 10)	*Rapana venosa* (Valenciennes, 1846)	8.43 ± 0.40 *	129.04 ± 18.40	Fishing, Black Sea
Mediterranean mussel—b (n = 10)	*Mytilus galloprovincialis* (Lamarck, 1819)	7.00 ± 0.26	19.71 ± 0.31	Fishing, Black Sea
Whiteleg shrimp—a (n = 10)	*Peneus vannamei* (Boone, 1931)	14.75 ± 1.38	22.11 ± 5.54	Aquaculture, Ecuador
Whiteleg shrimp—b (n = 10)	*Peneus vannamei* (Boone, 1931)	10.26 ± 2.25	24.55 ± 0.37	Aquaculture, Ecuador
Common octopus—cephalopod—b (n = 10)	*Octopus vulgaris* (Cuvier, 1797)	61.00 ± 1.41	1052 ± 0.01	Fishing, Atlantic Ocean Eastern Central
Mediterranean mussel (n = 10)—b	*Mytilus galloprovincialis* (Lamarck, 1819)	6.24 ± 0.79	11.16 ± 3.82	Aquaculture, Italy
Mediterranean mussel—a (n = 10)	*Mytilus galloprovincialis* (Lamarck, 1819)	7.16 ± 0.28	14.03 ± 2.57	Aquaculture, Spain
Chilean mussel—b (n = 10)	*Mytilus chilensis* (Hupé, 1854)	NA **	NA **	Aquaculture, Chile
Jumbo flying squid—a cephalopod (n = 10)	*Dosidicus gigas* (D’Orbigny, 1835)	NA **	NA **	Fishing, South-East Pacific Ocean
Indian squid—a cephalopod (n = 10)	*Uroteuthis duvaucelii* (D’Orbigny, 1835)	NA **	NA **	Fishing, West Indian Ocean
Webfoot octopus—b cephalopod (n = 10)	*Amphioctopus membranaceus* (Quoy & Gaimard, 1832)	NA **	NA **	Fishing, West Indian Ocean

* In case of the purple whelk, the height of the specimen was measured. ** not applicable because the biological material was purchased frozen and packed decorticated; a pre-boiled; b raw.

**Table 2:** Recovery rates (%) for the analyzed reference material.

Cd	Co	Cr	Cu	Fe	Mn	Ni	Pb	Zn
92.26	85.71	93.15	85.62	90.06	85.04	96.33	91.55	92.26

**Table 3:** Concentration levels of heavy metals (mean ± SD) in the muscle tissue of analyzed species, expressed as µg g^−1^.

Species	Cd		Co		Cr		Cu		Fe		Mn		Ni		Pb		Zn	
	Mean	SD	Mean	SD	Mean	SD	Mean	SD	Mean	SD	Mean	SD	Mean	SD	Mean	SD	Mean	SD
*M. chilensis_C1*	0.67	0.52	0.05	0.03	0.42	0.14	0.98	0.34	27.86	13.99	1.33	0.54	0.28	0.09	0.13	0.02	19.32	14.10
*M. chilensis_C2*	0.10	0.01	0.02	0.00	0.23	0.02	0.72	0.03	16.95	0.13	0.85	0.01	0.22	0.01	0.14	0.02	5.05	0.07
*M. galloprovincialis_S*	0.20	0.03	0.06	0.01	0.44	0.10	1.20	0.20	29.94	2.24	1.87	0.25	0.30	0.10	0.36	0.06	20.89	8.53
*M. galloprovincialis_I*	0.14	0.06	0.12	0.12	0.34	0.05	0.60	0.16	32.02	8.28	1.93	0.61	0.67	0.51	0.49	0.28	17.59	22.32
*M. galloprovincialis_BS*	0.09	0.04	0.03	0.00	0.26	0.02	0.59	0.26	19.78	0.90	1.55	0.24	0.29	0.02	0.18	0.01	11.12	5.69
*M. gigas*	0.09	0.02	0.05	0.01	0.33	0.02	23.29	16.71	26.14	6.74	4.28	3.03	0.16	0.01	0.21	0.04	96.94	48.62
*R. venosa*	0.05	0.01	0.03	0.00	0.51	0.05	22.74	7.57	77.80	16.20	1.53	0.07	0.35	0.01	0.16	0.02	9.50	1.21
*O. vulgaris_T*	0.31	0.06	0.02	0.00	0.31	0.02	4.99	2.23	12.23	0.08	0.61	0.03	0.92	0.34	0.27	0.04	14.68	0.62
*O. vulgaris_H*	0.72	0.36	0.03	0.00	0.35	0.03	8.60	1.22	20.38	6.18	0.60	0.05	0.93	0.42	0.26	0.04	13.33	0.49
*A. membranaceus_T*	0.49	0.60	0.03	0.01	0.31	0.03	1.99	0.40	14.21	1.56	0.56	0.27	0.43	0.06	0.14	0.01	7.67	1.16
*A. membranaceus_H*	0.55	0.26	0.03	0.01	0.29	0.04	2.39	0.73	21.46	9.00	0.59	0.14	0.43	0.11	0.13	0.02	8.17	1.02
*D. gigas*	0.06	0.01	0.01	0.00	0.25	0.01	0.80	0.32	9.08	0.65	0.24	0.03	0.26	0.03	0.10	0.01	8.36	0.69
*U. duvaucelli*	0.05	0.00	0.01	0.00	0.24	0.02	1.05	0.04	12.37	0.26	0.45	0.04	0.37	0.03	3.02	0.17	5.61	0.31
*P. vannamei_P*	0.02	0.00	0.03	0.02	0.64	0.35	3.12	0.86	27.32	17.91	0.74	0.48	0.49	0.27	0.13	0.03	7.88	0.62
*P. vannamei_R*	0.01	0.00	0.02	0.00	0.29	0.06	4.24	0.72	26.60	12.32	0.76	0.22	0.34	0.03	0.13	0.02	7.25	0.26

**Table 4:** Maximum levels for certain contaminants in foodstuffs, based on (EU) 2023/915.

Species	Pb	Cd
Crustaceans	0.5	0.5
Bivalve mollusks	1.5	1
Cephalopods	0.3	1

**Table 5:** Element concentrations in fresh seafood analyzed using TXRF.

Sample	P (mg g^−1^)	S (mg g^−1^)	K (mg g^−1^)	Ca (mg g^−1^)	Se (µg g^−1^)	As (µg g^−1^)
*A. membranaceus_H*	0.26 ± 0.01	1.02 ± 0.05	0.10 ± 0.00	1.04 ± 0.03	0.31 ± 0.00	0.35 ± 0.06
*A. membranaceus_T*	0.32 ± 0.01	1.08 ± 0.05	0.14 ± 0.01	0.80 ± 0.05	0.36 ± 0.00	0.3 ± 0.18
*M. chilensis*	1.44 ± 0.08	2.88 ± 0.22	2.29 ± 0.12	0.89 ± 0.03	1.68 ± 0.07	6.39 ± 0.27
*M. chilensis*	2.94 ± 0.04	2.78 ± 0.11	2.88 ± 0.11	0.70 ± 0.04	0.60 ± 0.02	3.21 ± 0.16
*M. chilensis*	2.24 ± 0.02	3.35 ± 0.06	2.86 ± 0.05	0.86 ± 0.01	0.82 ± 0.03	3.88 ± 0.03
*M. galloprovincialis*	2.80 ± 0.10	6.56 ± 0.35	6.50 ± 0.01	0.97 ± 0.04	0.95 ± 0.04	10.2 ± 0.25
*M. galloprovincialis*	1.07 ± 0.02	3.21 ± 0.07	3.41 ± 0.15	0.82 ± 0.01	0.90 ± 0.02	5.73 ± 0.34
*U. duvaucelli*	0.65 ± 0.02	1.63 ± 0.14	0.51 ± 0.00	0.97 ± 0.00	0.35 ± 0.03	1.16 ± 0.08
*M. gigas*	0.51 ± 0.01	2.51 ± 0.04	2.12 ± 0.09	2.16 ± 0.01	0.21 ± 0.01	5.66 ± 0.21
*O. vulgaris_H*	1.08 ± 0.02	3.13 ± 0.01	1.31 ± 0.04	0.94 ± 0.02	0.33 ± 0.00	5.47 ± 0.13
*O. vulgaris_T*	0.99 ± 0.03	3.08 ± 0.23	1.16 ± 0.05	0.76 ± 0.02	0.31 ± 0.02	4.19 ± 0.28
*M. gigas*	0.50 ± 0.02	2.31 ± 0.14	2.59 ± 0.05	0.63 ± 0.01	0.17 ± 0.01	7.14 ± 0.28
*R. venosa*	0.59 ± 0.02	1.85 ± 0.04	2.88 ± 0.05	1.84 ± 0.01	0.13 ± 0.00	1.29 ± 0.23
*R. venosa*	0.94 ± 0.06	2.45 ± 0.18	4.47 ± 0.20	1.78 ± 0.11	0.27 ± 0.00	1.92 ± 0.07
*P. vannamei*	1.14 ± 0.01	2.68 ± 0.09	5.40 ± 0.35	0.90 ± 0.01	0.44 ± 0.00	0.54 ± 0.02

**Table 6:** Estimated Daily Intake (EDI), target hazard quotient (THQ), and Hazard Index (HI) of heavy metals associated with several seafood species.

Species	Cd		Cr		Cu		Ni		Pb		Zn		HI
	EDI	THQ	EDI	THQ	EDI	THQ	EDI	THQ	EDI	THQ	EDI	THQ	
*A. membranaceus_H*	0.013	1.30 × 10^−5^	0.070	2.30 × 10^−5^	0.356	9.00 × 10^−6^	0.083	8.00 × 10^−6^	0.051	1.50 × 10^−5^	0.498	2.00 × 10^−6^	0.00007
*A. membranaceus_T*	0.024	2.40 × 10^−5^	0.056	1.90 × 10^−5^	0.448	1.10 × 10^−5^	0.079	7.00 × 10^−6^	0.035	1.00 × 10^−5^	0.515	2.00 × 10^−6^	7.25 × 10^−5^
*M. chilensis_S1*	0.022	2.20 × 10^−5^	0.087	2.90 × 10^−5^	0.148	4.00 × 10^−6^	0.110	1.00 × 10^−5^	0.033	9.00 × 10^−6^	0.767	3.00 × 10^−6^	7.65 × 10^−5^
*M. chilensis_S2*	0.001	1.00 × 10^−6^	0.313	1.04 × 10^−4^	0.182	5.00 × 10^−6^	0.097	9.00 × 10^−6^	0.038	1.10 × 10^−5^	0.711	2.00 × 10^−6^	0.00013
*M. chilensis_S3*	0.047	4.70 × 10^−5^	0.068	2.30 × 10^−5^	0.142	4.00 × 10^−6^	0.087	8.00 × 10^−6^	0.032	9.00 × 10^−6^	0.721	2.00 × 10^−6^	9.2 × 10^−5^
*M. galloprovincialis_S1*	0.002	2.00 × 10^−6^	0.104	3.50 × 10^−5^	0.077	2.00 × 10^−6^	0.106	1.00 × 10^−5^	0.037	1.10 × 10^−5^	1.117	4.00 × 10^−6^	6.3 × 10^−5^
*M. galloprovincialis_S2*	0.000	0.00	0.073	2.40 × 10^−5^	0.076	2.00 × 10^−6^	0.078	7.00 × 10^−6^	0.032	9.00 × 10^−6^	1.908	6.00 × 10^−6^	4.9 × 10^−5^
*U. duvaucelli*	BDL	BDL	0.177	5.90 × 10^−5^	0.138	3.00 × 10^−6^	0.044	4.00 × 10^−6^	0.031	9.00 × 10^−6^	0.205	1.00 × 10^−6^	7.2 × 10^−5^
*M. gigas_S1*	0.001	1.00 × 10^−6^	0.321	1.07 × 10^−4^	7.886	1.97 × 10^−4^	0.077	7.00 × 10^−6^	0.038	1.10 × 10^−5^	9.252	3.10 × 10^−5^	0.00035
*M. gigas_S2*	0.004	4.00 × 10^−6^	0.094	3.10 × 10^−5^	10.157	2.54 × 10^−4^	0.059	5.00 × 10^−6^	0.030	9.00 × 10^−6^	12.679	4.20 × 10^−5^	0.0003
*O. vulgaris_H*	0.005	5.00 × 10^−6^	0.199	6.60 × 10^−5^	0.400	1.00 × 10^−5^	0.078	7.00 × 10^−6^	0.033	1.00 × 10^−5^	0.673	2.00 × 10^−6^	0.0001
*O. vulgaris_T*	0.001	1.00 × 10^−6^	0.087	2.90 × 10^−5^	0.359	9.00 × 10^−6^	0.067	6.00 × 10^−6^	0.030	9.00 × 10^−6^	0.509	2.00 × 10^−6^	5.6 × 10^−5^
*R. venosa*	BDL	BDL	0.084	2.80 × 10^−5^	0.684	1.70 × 10^−5^	0.127	1.20 × 10^−5^	0.035	1.00 × 10^−5^	0.757	3.00 × 10^−6^	9.4 × 10^−5^
*R. venosa*	BDL	BDL	0.068	2.30 × 10^−5^	2.135	5.30 × 10^−5^	0.103	9.00 × 10^−6^	0.031	9.00 × 10^−6^	0.733	2.00 × 10^−6^	5.3 × 10^−5^
*P. vannamei*	BDL	BDL	0.070	2.30 × 10^−5^	0.520	1.30 × 10^−5^	0.103	9.00 × 10^−6^	0.037	1.10 × 10^−5^	0.333	1.00 × 10^−6^	6.6 × 10^−5^

**Table 7:** Target Cancer Risk of heavy metals associated with several seafood species.

Species	TR			
	Cd	Cr	Ni	Pb
*A. membranaceus_H*	4.85 × 10^−9^	3.48 × 10^−8^	1.41 × 10^−7^	4.34 × 10^−10^
*A. membranaceus_T*	9.11 × 10^−9^	2.78 × 10^−8^	1.34 × 10^−7^	2.94 × 10^−10^
*M. chilensis*	8.3 × 10^−9^	4.35 × 10^−8^	1.87 × 10^−7^	2.81 × 10^−10^
*M. chilensis*	2.42 × 10^−10^	1.56 × 10^−7^	1.65 × 10^−7^	3.25 × 10^−10^
*M. chilensis*	1.78 × 10^−8^	3.4 × 10^−8^	1.48 × 10^−7^	2.69 × 10^−10^
*M. galloprovincialis*	9.01 × 10^−10^	5.22 × 10^−8^	1.81 × 10^−7^	3.14 × 10^−10^
*M. galloprovincialis*	1.04 × 10^−10^	3.65 × 10^−8^	1.33 × 10^−7^	2.71 × 10^−10^
*U. duvaucelli*	BDL	8.84 × 10^−8^	7.46 × 10^−8^	2.6 × 10^−10^
*M. gigas*	4.18 × 10^−10^	1.6 × 10^−7^	1.31 × 10^−7^	3.23 × 10^−10^
*O. vulgaris_H*	2.01 × 10^−9^	9.93 × 10^−8^	1.32 × 10^−7^	2.83 × 10^−10^
*O. vulgaris_T*	5.36 × 10^−10^	4.36 × 10^−8^	1.14 × 10^−7^	2.55 × 10^−10^
*M. gigas*	1.36 × 10^−9^	4.72 × 10^−8^	1.01 × 10^−7^	2.58 × 10^−10^
*R. venosa*	BDL	4.18 × 10^−8^	2.16 × 10^−7^	2.99 × 10^−10^
*R. venosa*	BDL	3.42 × 10^−8^	1.75 × 10^−7^	2.67 × 10^−10^
*P. vannamei*	BDL	3.48 × 10^−8^	1.75 × 10^−7^	3.17 × 10^−10^

**Table 8:** Metal bioaccessibility from various studies.

Fish/Seafood Sample	Cooked/Raw	Studied Element	Metal Bioaccessibility (%)	Reference
Cod	Cooked	Selenium	61	[35]
Tuna	Cooked	Mercury	6–10	[18]
Tuna	Raw	Mercury	13–19	[18]
Swordfish	Raw	Mercury	59–87	[18]
Swordfish	Cooked	Mercury	38–49	[18]
*Tope shark*	Raw	Mercury	59–69	[18]
*Tope shark*	Cooked	Mercury	34–47	[18]
*Butter clams*	Raw	Cadmium	107	[22]
		Arsenic	108	
		Mercury	50	
		Selenium	98	
		Manganese	102	
		Copper	117	
Salmon eggs	Raw	Cadmium	74	[22]
		Arsenic	73	
		Mercury	10	
		Selenium	76	
		Manganese	96	
		Copper	106	
*Chinook salmon*	Raw	Cadmium	18	[22]
		Arsenic	57	
		Mercury	49	
		Selenium	52	
		Manganese	38	
		Copper	64	
*Sockeye salmon*	Raw	Cadmium	61	[22]
		Arsenic	68	
		Mercury	46	
		Selenium	50	
		Manganese	48	
		Copper	63	
Seaweed	Raw	Cadmium	64	[22]
		Arsenic	79	
		Mercury	-	
		Selenium	65	
		Manganese	86	
		Copper	59	
*Rapa whelk*	Raw	Chromium	24.27–57.14	[10]
		Manganese	17.47–32.43	
		Copper	70.97–83.56	
		Zinc	58.21–71.12	
		Cadmium	61.58–79.46	
		Nichel	3.26–71.48	
Seabass	Raw	Arsenic	81.2–90.8	[55]
	Cooked		73.3–86.2	
	Raw	Cadmium	84.8–93.2	
	Cooked		36.2–86.5	
	Raw	Copper	81.4–84.2	
	Cooked		54.2–71.2	
	Raw	Iron	51.3–58.0	
	Cooked		27.7–53.7	
	Raw	Selenium	60.8–61.0	
	Cooked		29.0–62.2	
	Raw	Zinc	70.4–72.3	
	Cooked		36.8–52.2	
Red seabream	Raw	Arsenic	74.0–87.9	[55]
	Cooked		3.7–86.5	
	Raw	Cadmium	73.7–89.9	
	Cooked		52.2–88.0	
	Raw	Copper	82.7–85.4	
	Cooked		58.2–71.2	
	Raw	Iron	45.4–52.0	
	Cooked		24.6–53.2	
	Raw	Selenium	48.0–63.6	
	Cooked		27.4–66.2	
	Raw	Zinc	66.6–73.1	
	Cooked		28.1–51.5	
Shellfish—*C. ariakensis*	Raw	Zinc	16.24–22.52	[8]
		Copper	42.88–6.38	
		Cadmium	12.59–33.22	
		Arsenic	49.52–69.23	
		Chromium	65.41–66.36	
		Lead	22.96–73.64	
Shellfish—*C. farreri*	Raw	Zinc	21.95–48.80	[8]
		Copper	34.63–70.13	
		Cadmium	11.42–45.41	
		Arsenic	61.49–79.31	
		Chromium	58.72–66.54	
		Lead	45.90–82.35	
Shellfish—*S. constricta*	Raw	Zinc	17.26–3.7.01	[8]
		Copper	32.79–49.88	
		Cadmium	12.31–29.11	
		Arsenic	28.69–61.68	
		Chromium	56.72–59.88	
		Lead	44.62–72.36	
Yellow croaker	Raw	Arsenic	87.4–98.4	[56]
Snapper	Raw	Arsenic	98.4	
Turbot	Raw	Hg	96.8	
Clam	Raw	Hg	64.7	
Turbot	Raw	Cadmium	60.0–99.4	
Turbot	Raw	Lead	78.9–93.8	
Turbot	Raw	Nichel	75.9–94.3	
Snapper	Raw	Chromium	20.2	
Clam	Raw	Chromium	87.6	
Seafood	Cooked	Mercury	14–39	
Seafood	Cooked	Arsenic	2.7–35.2	
Black scabbardfish Black scabbardfish	Raw	Mercury	40	[57]
	Cooked	Mercury	10	
Mollusks	Raw	Arsenobetaine	100	[58]
Shellfishes	Cooked	Arsenic	Gastric: 40.9, Intestinal: 52.5	
*Chlamys farreri*-mantle	Raw	Cadmium	11.2–49.5	[59]
*Chlamys farreri*-mantle	Cooked	Cadmium	9.4–27.1	
Octopus	Raw	Mercury	11	[54]
	Cooked		1	
Hake	Raw	Mercury	61	
	Cooked		19	
Octopus		Arsenic	98	
Mussel	Raw	Cadmium	103	
	Cooked		83	
Shrimp	Raw	Cadmium	75	
	Cooked		54	
Tuna	Raw	Cadmium	41	
	Cooked		53	
Shrimp	Raw	Selenium	93	
	Cooked		74	
Octopus	Raw	Copper	93	
	Cooked		100	
Octopus	Raw	Manganese	113	
Mussel	Raw	Iron	26	
	Cooked		13	

## Data Availability

The original contributions presented in this study are included in this article. Further inquiries can be directed to the corresponding authors.
